# Addition of Amaranth Flour of Different Particle Sizes at Established Doses in Wheat Flour to Achieve a Nutritional Improved Wheat Bread

**DOI:** 10.3390/foods12010133

**Published:** 2022-12-27

**Authors:** Ionica Coțovanu, Silviu-Gabriel Stroe, Florin Ursachi, Silvia Mironeasa

**Affiliations:** Faculty of Food Engineering, Stefan cel Mare University of Suceava, 720229 Suceava, Romania

**Keywords:** wheat–amaranth composite flour, amino acids, minerals, dough rheology, bread, sensory properties, nutritional profile

## Abstract

Amaranth is an underutilized pseudocereal that can be used to supplement wheat flour (WF) in order to improve the nutritional quality of bread. Bread digestibility is impacted by particle size which produces different nutritional properties. This research aims to evaluate the baking characteristics of optimal wheat–amaranth composite flour for each studied amaranth flour (AF) particle size at doses previously established based on an optimization process and to characterize from a physical, textural, nutritional, and sensorial point of view the obtained bread. The results revealed that the optimal wheat–amaranth composite flour with medium and small particle size, respectively showed a slightly lower α-amylase activity, while dough development time was significantly higher compared to the WF. A significant (*p* < 0.05) decrease was observed in the elasticity, deformation energy, and dynamic rheological parameters of the optimal composite dough for all the particle sizes, whereas fermentation parameters showed higher values compared to the control, indicating the ability of the gluten structure in large, medium, and small particle sizes of AF to hold the gas and to expand without collapsing. The physical, textural, and especially nutritional characteristics of the optimal WF-AF bread were enhanced. The sensory evaluation results revealed high scores (8.70) for the acceptability of optimal bread with a medium particle size as compared to wheat bread (8.25). The protein and ash content of the optimal breads with large, medium, and small AF particle sizes, respectively, increased significantly, from 8.92 to 10.58%, and 0.82 to 0.99%, respectively, relative to the wheat flour bread (8.35% and 0.72%, respectively). The mineral content was up to two times higher in the optimal breads compared to wheat flour bread. The findings generated from this study are helpful in bakery industry for designing products with enhanced nutritional properties and for introducing new products to the market.

## 1. Introduction

Bread is one of the most frequently consumed foods, being the main source of energy supplied for the body [1], but refined wheat flour, which is the key ingredient for the manufacture of bread and bakery products, has a reduced nutritional value due to its lower contents of fiber, vitamins, minerals, and other compounds compared to whole wheat flour [2]. Numerous studies in the specialized literature highlight the improvement of the nutritional characteristics of wheat flour through the incorporation of new functional ingredients and the development of safe and healthy nutritional products [3,4,5]. In this sense, bakery products enriched with dietary fiber, amino acids, and bioactive compounds from flours obtained from whole grains or pseudo-cereals, which prevent the diseases associated with the metabolic syndrome, such as cardiovascular diseases, arteriosclerosis, and colon cancer, are an alternative [6].

Interest in pseudocereals’ use in food technologies has increased in recent years, because they offer a valuable nutritional composition, having a high-quality protein content amino acid composition point of view [7]. Amaranth is a pseudocereal recognized for its main functional components, namely dietary fiber (11.10%) [8], proteins (10.18–29.35%) [9], polyunsaturated fatty acids (3.23%) [10], vitamins (riboflavin: 0.19–0.23%), ascorbic acid (4.50%), folic acid and vitamin E [11], minerals (calcium: 178 mg/100 g; magnesium: 248 mg/100 g; phosphorus: 557 mg/100 g; potassium: 508 mg/100 g) [10], and various bioactive compounds [12]. The biggest limitation in the functional properties is that amaranth does not contain gluten and therefore has no dough-forming or baking properties. Due to the lack of gluten in amaranth, its addition to flour, especially in large quantities, in wheat flour dough results in a modification of the processing conditions and the quality of the final product [11,13,14]. Up to a certain amount, amaranth flour can be added to wheat-based products, thereby improving the nutritional properties of the resulting product. Depending on the amount of wheat flour substituted (12–50%), several groups of researchers have reported an improvement of the final product compared to wheat flour bread in terms of protein (from 14.29% to 16.30%), lipids (from 0.67% to 1.75%), fiber, and minerals (in particular, Cu: from 2.25 to 4.21 µg/g, Mn: from 6.39 to 19.41 µg/g, Zn: from 11.65 to 24.95 µg/g, Fe: from 18.85 to 43.74 µg/g, Ca: from 0.31 to 0.99 mg/g, Mg: from 0.29 to 1.32 mg/g, and K: from 1.88 to 3.21 mg/g) [13,14,15]. Improved bakery products can be formulated by adding amaranth flour to refined wheat flour, at different addition doses and particle sizes, with specific functional properties. The functionality and chemical composition of flours are influenced by their particle size distribution. Fractions of flour with different particle sizes sieved from the same flour result in different chemical compositions [9], which will affect the wheat–amaranth flour’s composition [16]. The incorporation of amaranth flour in bread-making may influence dough rheological properties and consequently the baking performance of bread [17,18]. Rosales-Juárez et al. [19] demonstrated that specific bread volumes, bread colors, textural properties, and flavors are correlated with WF quality and the breadmaking processes. For example, the gluten in wheat flour is vital for the viscoelastic properties and carbon dioxide retention capacity of bread dough during fermentation and the initial stages of baking [20,21] and consequently determines bread volume [22] and textural properties of breadcrumbs [23].

The use of protein-rich and gluten-free amaranth flour mixed with cereal flour had a negative impact on the dough’s rheological properties. The addition of amaranth flour at different doses (10, 20, and 30%) in refined wheat flour or whole wheat flour determined an increase in dough tenacity and a decrease in its extensibility [24]. In another study [25], it was found that bread enriched with expanded amaranth in concentrations of 10, 15 and 20% had a higher content of minerals (Zn, Cu, Fe, Mn, Na, K, Mg, and Ca), proteins (13.90%), dietary fiber (1.11%), and squalene (43 mg/100 g), but the bread’s specific volume decreased by up to 33%. The addition of *Amaranthus cruentus* whole meal in gradual doses up to 40% to wheat flour remarkably increased the protein, lipid, ash, and dietary fiber contents of white bread [13]. An increase in minerals such as Cu, Mn, Zn, Fe, Ca, Mg, and P was also found, while the hardness of the crumb was influenced only by the sample containing 40% amaranth, and the structure of the crumb did not show significant changes. An improvement of the nutritional value of the product by increasing the content of dietary fiber, minerals, and proteins was reported when whole wheat flour was substituted with amaranth at 50% [26]. Although, amaranth bread did not achieve higher acceptability than wheat bread, especially the one with a high percentage of substitution (40%), consumers concluded that the preference for amaranth bread is based on its nutritional value, even if the taste and flavor are different from wheat bread.

Enhancing nutritional aspects when using wheat–amaranth composite flour with different particle sizes of amaranth in bread-making requires an adapted formulation. A desirability function has previously been applied to find the optimal amount of AF added in WF, depending on the AF particle size, to enhance dough and bread properties [27]. These optimal doses consider the baking characteristics of the flour, and bread quality parameters were established based on predictive models obtained for twenty-four parameters, as shown in a previous study [27], by applying the analysis of multiple responses. It was highlighted that AF of large, medium, and small particle size added to WF at different doses is crucial for obtaining the desired rheological properties (determined by using empirical and dynamic rheological tests) and a suitable bread volume and textural firmness. However, there are no studies that report the effects of optimal wheat–amaranth composite flour depending on different amaranth flour particle sizes in terms of the physical properties, degree of liking (consumer acceptability), and nutritional profile of the resulting bread. Therefore, the aim of this study was to evaluate the chemical, physical, textural, and sensory properties of bread baked from optimal wheat–amaranth composite flour specific to large, medium, and small amaranth flour particle size.

## 2. Materials and Methods

### 2.1. Raw Materials

Amaranth seeds were purchased from Solaris Plant (Ilfov, Romania), wheat flour (WF) (flour yield 65%) from Mopan (Suceava, Romania), fresh *Saccharomyces cerevisiae* yeast from Rompak (Paşcani, Romania), and salt from SanoVita (Vâlcea, Romania).

### 2.2. Wheat–Amaranth Flour Preparation

The wheat flour type 650 with a wet gluten content of 30.00%, dry gluten content of 9.75%, gluten deformation index of 6 mm, and FN index of 312 s was used in the research. The bread evaluated in this study was made from wheat–amaranth composite flour, formulated based on the optimal amounts obtained for each particle size in previous research [28]. The procedure used for amaranth flour (AF) preparation in order to obtain large, AL (>300 µm, <500 µm), medium, AM (>180 µm, <300 µm) and small, AS (<180 µm) particle size was described in previous studies [9,27]. For the optimal wheat–amaranth composite flour formulations (O_AL, O_AM, and O_AS), amaranth flour was added to WF at 9.41, 9.39, and 7.89% from large, medium, and small particle sizes, respectively, and mixed for 30 min in a Yucebas Y21 machine (Yücebaş Makine, Izmir, Turkey). Wheat flour (WF) was considered as the control.

### 2.3. Dough and Bread Making Preparation

Bread dough samples were prepared from wheat flour and optimal composite flours using the indirect method, at the optimum water absorption capacity determined at Mixolab (Chopin, Tripetteet Renaud, Paris, France), according to the method of Coțovanu and Mironeasa [27]. For the evaluation of the empirical and dynamic rheological properties, the dough samples were prepared without yeast, except the dough used for the rheofermentographic evaluation. Texture profile analysis was applied to dough with yeast to evaluate his behavior during processing. The raw materials used to obtain the bread were optimal wheat–amaranth composite flour (300 g), salt (1.8%), and *Saccharomyces cerevisiae* baker’s yeast (3%). The biphasic procedure for the preparation of dough samples consisted in mixing half of the composite flour amount with the entire amount of water and yeast in order to form a leaven 30 ± 2 °C and 85% relative air humidity (RH). The leaven thus formed was left to ferment for two hours in a fermentation chamber (PL2008, Piron, Cadoneghe, Padova, Italy). After the leaven had fermented for two hours, the other part of optimal composite flour and salt were incorporated, kneaded for 10 min using a Kitchen Aid mixer (Whirlpool Corporation, Benton Harbor, MI, USA), and allowed to complete its process of sugar fermentation for another hour at 30 ± 2 °C and 85% RH [9]. The dough was then divided into 400 g pieces, which were shaped by hand and left to rise on aluminum trays for one hour (30 ± 2 °C and 85% RH). The leavened dough was baked in the oven (Caboto PF8004D, Cadoneghe, Padova, Italy) at 220 ± 5 °C for 25 ± 2 min. Bread from 100% wheat flour (WFB) was used as the control. After baking the bread samples were left at room temperature for two hours to cool, and then the physical, textural and sensorial determinations were made.

### 2.4. Proximate Analysis

The bread samples were analyzed for moisture content according to the gravimetric method; the protein content was determined using a rapid Kjeldahl device with digestion and steam distillation (VELP Scientifica, Usmate Velate, Italy), and the conversion factor was 6.25 for WFB and 5.53 for composite flour bread. The ash content was determined in a muffle oven by incineration at 900 °C, per the standard protocols (110/1, 105/2, 136, 104/1) of the International Association for Cereal Chemistry [29], while the carbohydrate content was calculated by difference (% of dry matter). The energy values (kcal) were calculated using conversion factors according to EU Regulation No 1169/2011 (4.1 kcal per g for protein and sugars, and 9.3 kcal per g for lipids). Each analysis was conducted at least in duplicate.

### 2.5. Determination of Mineral Content by Atomic Absorption Spectrometry

Atomic absorption spectrometry (AAS) was used to identify chemical elements through the absorption of optical radiation by a population of free atoms in the gaseous state. For the determination of macro- and micro-elements from wheat flour, amaranth fractions, and bread, the method described in the SR EN 14082:2003 standard was used. The analysis of the sample involved two stages: the mineralization of the sample and the metal dosage by spectrophotometry. During mineralization, the organic matter in the sample (5.00 ± 0.001 g) was destroyed by carbonization and combustion in the calciner, with the temperature gradually increasing from 250 °C to 450 °C, up to 900 °C, for 8 h. A total of 5 mL HCl 6 mol/L (STAS 13013/1-91) was added to the ash obtained, and then the acid was evaporated using a sand bath. The residue was dissolved with 730 µL HNO_3_ 69% and brought to the mark (50 mL) with deionized water. As a control sample, deionized water was used following the same procedure as for the analyzed sample. The spectrophotometric determination involved the following steps: activating the cathode lamp corresponding to the elements (K, Ca, Mg, Na, Fe, Zn, Mn, and Cu), adjusting the operational parameters, activating and adjusting the flame, and establishing the curve standard by absorbing four working standard solutions of different concentrations. In the flame system used, the nebulizer and the atomizer play a decisive role: the nebulizer aspirates a liquid sample with a controlled flow and the atomizer creates a fine aerosol and mixes the aerosol with the oxidizing gas. The mineral elements are expressed as mg/100 g of flour and were calculated with Equation (1):

(1) $$E=\frac{C\cdot F\cdot V}{M}$$

where:

E—mineral element concentration, mg/100 g;
C—the concentration measured on the calibration curve, mg/L;
F—dilution factor;
V—sample volume, mL;
M—sample mass taken in the analysis, g.

### 2.6. Amino Acid Content Determination

In order to determine the amino acid (AA) content of wheat flour, amaranth fractions, and bread, first the samples (3.70 ± 0.5 g) were mixed with 30 mL of trichloroacetic acid (TCA) 15%. Next, the pH of the solution was adjusted to 2.2 with sodium hydroxide solution and further diluted with 50 mL of 15% TCA. After centrifugation for 5 min at 3000 rpm, the supernatant was filtered through a 0.45 µm filter [30]. The solution contained primary and secondary amino acids that were further analyzed with the Ez:faast GC-MS kit (Phenomenex, Torrance, CA, USA). Amino acid analysis was performed with a gas chromatograph (GC) coupled to a mass spectrometer (Shimadzu, Kyoto, Japan). The analysis time was 10 min, and the injected volume was set to 0.002 mL. Separation of the amino acids was performed in a ZB-AAA column (10 m × 0.25 mm). The split-less injection mode was applied. The initial temperature of the GC oven was 110 °C, which was increased to 320 °C and held for three min. The temperature conditions used for the mass spectrometer were 200 °C for the ion source and 320 °C for the interface. The quadrupole measured ion abundances from 35 to 500 m/z. Amino acid mixture solutions included in the kit were used for calibration [31]. Seven essential amino acids were determined for the samples taken in the study: isoleucine, leucine, methionine, phenylalanine, threonine, tryptophan, and valine. Eleven non-essential amino acids were determined for amaranth fractions and wheat flour: alanine, aspartic acid, glutamic acid, glutamine, glycine, serine, tyrosine, asparagine, proline, thioproline, and hydroxyproline

### 2.7. Baking Characteristics Determination of Flour Samples Formulated

#### 2.7.1. Dynamic Rheological Tests

A Thermo-HAAKE, MARS 40 (Karlsruhe, Germany) with parallel plate-plates geometry was used to determine the dough dynamic rheological behavior in terms of elastic (G′) and viscous modulus (G″), viscosity factor (tan δ), maximum gelatinization temperature (T_max_), and creep-recovery compliance (Jc_max_, Jr_max_) [27].

#### 2.7.2. Empirical Rheological Tests

The falling number index (FN) of the wheat flour and optimal composite flour related to each of the studied particle size of AF was determined using a Falling Number device (FN 1305, Perten Instruments AB, Stockholm, Sweden) [27].

The Mixolab Chopin equipment (Tripette et Renaud, Paris, France) was used for a complete rheological test of wheat flour and optimal wheat–amaranth composite flour formulations following the ICC 173, AACC 54–60.01 method [30]. The Mixolab parameters measured were water absorption, WA (%), dough development time, DT (min), the dough stability, ST (min), the protein weakening (C1-2), starch gelatinization (C3-2), starch breakdown (C3-4), and starch recrystallization (C5-4) [27].

The biaxial extension of dough in terms of dough tenacity (P), dough extensibility (L), dough strength (W), and alveograpic ratio (P/L), was analyzed with an Alveograph Chopin equipment (KPM Analytics, Villeneuve-la-Garenne, France) following the ICC 121 method [29].

Dough fermentation testing, in terms of maximum height of the gas release curve (H’m), the total volume of CO_2_ production (VT), the volume of the gas retained in the dough at the end of the test (VR), and retention coefficient (CR), was performed with a rheofermentometer device (Chopin Rheo, type F4, Villeneuve-La-Garenne, France), as described in a previous study [27], following the AACC 89–01.01 method [32].

### 2.8. Evaluation of Bread Physical and Textural Characteristics

The physical characteristics of bread samples made from wheat flour and optimal wheat–amaranth composite flour, bread volume (BV), specific volume, porosity, and elasticity was determined according to the Romanian standard SR 90: 2007 [33].

A TVT-6700 texture analyzer (Perten Instruments, Hägersten, Sweden) was the equipment used for analyzing bread firmness, elasticity, cohesiveness, gumminess, resilience, and masticability, by using the working setting shown in a previous study [27], and the values were recorded and processed using the TexCalc 5 software (5.1.0.x. version, Perten Instruments, Hägersten, Sweden).

### 2.9. Bread Colour Measurement

The CIELAB colour coordinates for the lightness (L*), redness/greenness (a*), and yellowness/blueness (b*) of the crust and crumb bread samples were measured with a CR-700 colorimeter (Konica Minolta, Tokyo, Japan).

### 2.10. Bread Sensorial Properties

The sensory characteristics of the bread were evaluated by semi-trained tasters from the Faculty of Food Engineering in Suceava (13 people aged between 20–55). The method consisted of identifying the most suitable terms to describe the general acceptability, appearance, crust and crumb structure, taste, and smell. The terms proposed in the sensory evaluation were those mentioned in the SR ISO 11035:2007 standard [34], which describes the sensory profile method. The overall acceptability of the samples was scored using a hedonic scale (1–9), with the highest score being given to the highest level of acceptance, and the lowest score to a decreased level of enjoyment.

### 2.11. Statistical Analysis

All the data in the tables and figures were presented as a mean with standard deviation, and the determinations were performed at least in duplicate. SPSS software 25.0 (trial version) (IBM, New York, NY, USA) was used for statistical analysis of the data. Statistically significant differences between samples were determined by one-way ANOVA. Tukey’s test was used to test the differences between the characteristics of the optimal and control samples. Pearson’s coefficient at *p* < 0.05 was used to test the relationships among the studied parameters. A principal component analysis (PCA) was applied to observe the relationships between the wheat–amaranth dough, bread proximate composition, textural characteristics, and acceptability, and to visualize similarities or dissimilarities between them.

## 3. Results

### 3.1. Nutritional Characteristics of Wheat Flour and Amaranth Flour Fractions

The physicochemical characterization of the composition of wheat flour and amaranth flour fractions was reported in a previous study [27]. In addition, in this work, the mineral and amino-acid contents were determined, and the results were presented in the Supplementary Materials (Table S1 and Figure S1).

### 3.2. The Baking Characteristics of the Optimal Wheat–Amaranth Composite Flour and the Quality of the Bread

The baking characteristics of the optimal wheat–amaranth formulated composite flours and the quality parameters of the bread compared to wheat dough and bread are shown in Table 1.

According to the obtained results, the optimal wheat–amaranth composite flours with large fractions presented α-amylase activity, quantified by the falling number (FN) index, similar to that of the control sample, whereas the optimal composite flour with medium and small fraction presented a slightly lower α-amylase activity.

Dough stability (ST), protein thermal weakening (C1-2), and starch gelatinization (C3-2) also showed values very close to those of the control sample, while the dough development time (DT) and the stability of the hot gel (C3-4) were considerably higher than the control sample, whilst starch retrogradation (C5-4) presented significantly higher values for the optimal composite flour than for wheat flour.

Alveographic parameters’ values presented differences between samples, the highest values of dough tenacity being observed for the optimal dough with small AF particle size, while the lowest extensibilty value was obtained for the optimal composite dough with a large particle size.

The optimal dough samples with large, medium, and small particle sizes had a higher maximum height (H’m), volume of gas total (VT) and retained (VR), and gas retention capacity (CR) compared to wheat flour dough. The elasticity (G′) and viscosity (G″) modulus showed higher values than the control for all of the optimal dough samples, which indicated the viscoelastic nature of the dough, the results being expected. Remarkable differences were obtained between the optimal wheat–amaranth composite flour dough samples with large fraction sizes (O_AL) and those with medium and small sizes (O_AM and O_AS). The maximum gelatinization temperature (T_max_) decreased compared to the control sample. A lower resistance to deformation indicated by an increase in the creep compliance (J_cmax_) value was obtained for the samples with the optimal addition of small sizes of amaranth flour compared to the control sample.

Regarding bread volume, an increase was observed in the optimal composite flour bread with medium particle size, being similar to that of the wheat flour bread, while the optimal composite bread with large and small particle sizes presented lower values for bread volume.

### 3.3. Advanced Characterization of the Bread Obtained from Optimal Wheat–Amaranth Composite Flour for Each Amaranth Flour Particle Size Studied

#### 3.3.1. The Physical Characteristics of Optimal Bread

According to the experimental data, significant differences were obtained between the specific volume, porosity, and elasticity of bread samples with the optimal addition levels of amaranth flour fractions (Table 2).

The optimized bread with the small fraction showed a lower specific volume than the control sample. Regarding the porosity and elasticity of the bread crumb with the optimal dose of amaranth flours, the best result was obtained for the large fraction.

Colour is an essential attribute of food products, and it influences consumer perception and food product acceptability. Flour was one of the factors that determined bread samples’ color. The colour parameters—the lightness (L*), the intensity of the red (a*) or green (−a*), and the yellow (b*) or blue (−b*) shade—for the crust and the crumb of the breads made from the optimal composite flours varied according to the related optimal dose (Table 3).

In the case of bread samples with the optimal dose of amaranth fractions, the crust lightness decreased in the following order: O_AL < O_AS < O_AM. The values of the crust bread *a* varied in the positive range for bread samples for optimal composite flour and in the negative range for the control sample, and the highest value was obtained for bread from the optimum flour composite with a medium particle size of the amaranth flour.

#### 3.3.2. Texture Parameters of the Optimal Breads

The textural parameters of bread have a direct influence on the consumer’s perception and choice, and the physical parameters of the bread samples obtained with the optimal dose of AF addition corresponding to the large (L), medium (M), and small (S) particle sizes compared to bread from refined wheat flour are presented in Table 4.

According to the experimental data obtained from the texture analysis of the bread samples, significant differences (*p* < 0.05) were observed between the firmness of bread crumb with AF fractions compared to the control. A gradual increase in firmness was observed in bread with particle size reduction, while the bread sample with large AF particles showed lower firmness values. The elasticity of the bread samples with the optimal dose of amaranth decreased in the following order: M < L < S, with significant differences between the optimal samples and the control bread, but no significant differences were recorded between the samples with medium and small particles.

#### 3.3.3. Nutritional Composition and Energy Value of Breads with Amaranth Flour at the Optimal Addition Dose for Each Studied Particle Size

The physico-chemical properties of the bread samples made from the optimal composite flours for each AF compared to the control bread are shown in Table 5.

The chemical composition of the optimized bread samples, corresponding to each amaranth flour particle size, showed significant differences (*p* < 0.05) between the samples and compared to the control sample. The results indicated an improvement in the protein, lipid, and ash contents of bread obtained from the optimal composite flour, especially in the case of large and medium fractions of amaranth flour, while the carbohydrate content and energy values decreased (Table 5).

#### 3.3.4. Macro- and Micro-Elements Content of the Optimal Bread Samples

**Figure 1:**
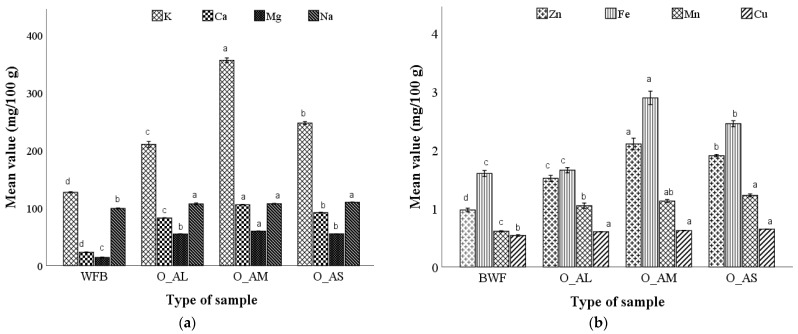
Macro- (**a**) and micro-mineral (**b**) composition of the bread with the optimal dose (O) of amaranth flour added corresponding to the large (L), medium (M), and small (S) particle sizes compared with control bread. Mean values followed by different letters (a–d) are significantly different (*p* < 0.05).

The composition in macro- and micro-elements of the bread samples obtained from the optimal composite flours was influenced by the particle size composition of the amaranth flour which partially replaced refined wheat flour (Figure 1). An increase in the content of macro- and micro-elements was observed depending on the particle size as follows: O_AM > O_AS > O_AL. The optimal bread samples obtained from wheat–amaranth composite flour presented a significantly higher macro and mineral content. The higher values of K (356 mg/100 g), Ca (105 mg/100 g), and Mg (59.7 mg/100 g) were found in the optimal bread with a medium amaranth flour fraction, while no differences in sodium content were found in the optimal composite bread samples, in contrast to the wheat flour bread where identified K (126 mg/100 g), Ca (23 mg/100 g), and Mg (13.7 mg/100 g) were identified. The optimal composite wheat–amaranth medium fraction bread contains 2.20 mg/100 g Zn, 3.00 mg/100 g Fe, 1.13 mg/100 g Mn, and 0.623 mg/100 g Cu, values that are significantly higher than those present in wheat flour bread (0.94, 1.54, 0.62, and 0.55, respectively). The mineral content of the optimal bread samples increased significantly as a result of wheat flour replacement, as expected due to the amaranth flour particle size composition.

#### 3.3.5. Determining the Amino Acid Content of Optimal Bread Samples

**Figure 2:**
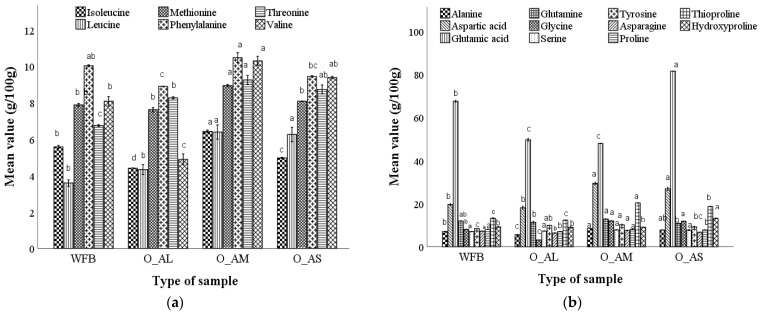
Essential (**a**) and non-essential (**b**) amino acid composition of the bread with the optimal dose (O) of amaranth flour added corresponding to the large (L), medium (M), and small (S) particle size compared to control bread. Mean values followed by different letters (a–d) are significantly different (*p* < 0.05).

The variation of the essential and non-essential amino acid (AA) content in bread samples with the optimal dose (O) of amaranth flour addition corresponding to large (L), medium (M), and small (S) particle sizes and in comparison with bread made from refined wheat flour is presented in Figure 2.

Six essential amino acids were identified in bread samples formulated with the optimal doses of amaranth flour, for each individual particle size, (Figure 2a), representing 16.16% for the bread with the large fraction, 15.3% for the bread with the medium fraction, and 13.48% for the bread with the small fraction of amaranth flour, from the total AA profile. The amino acid content for the bread samples formulated with the optimal doses of AF fractions varied as follows: isoleucine (4.41–6.35%), leucine (4.33–6.39%), methionine (7.62–8.94%), phenylalanine (8.9–10.48%), threonine (9.30–9.25%), valine (4.89–10.30%), alanine (5.60–8.53%), aspartic acid (18.12–29.45%), glutamic acid (4.97–81.47%), glutamine (109.00–115.24%), glycine (3.20–12.03%), serine (7.32–8.06%), tyrosine (9.25–10.25%), asparagine (6.43–7.64%), proline (7.05–8.15%), thioproline (12.37–20.38%), and hydroxyproline (9.06–13.25%). An increase in AA content was observed in bread with the optimal dose of medium particles, followed by the bread with small-size particles and a lower content than in the control sample was identified in bread obtained with the optimal dose of AF large particles.

#### 3.3.6. Sensory Analysis of Breads Obtained from Optimal Composite Flours

**Figure 3:**
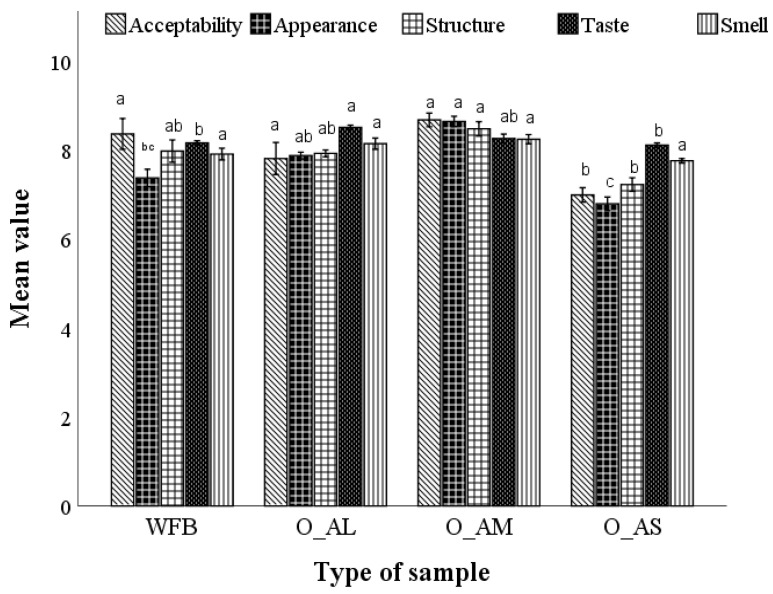
Sensory properties of bread samples with the optimal dose (O) of amaranth flour added corresponding to the large (L), medium (M), and small (S) particle size compared to wheat flour bread (WFB). Mean values followed by different letters (a–c) are significantly different (*p* < 0.05).

The results of the sensory analysis in terms of overall acceptability, appearance, crumb structure, taste, and smell for the optimal bread formulations compared to the control can be observed in Figure 3. From the average scores of the sensory characteristics evaluated in this study, it can be observed that the optimal bread with medium fractions was preferred by the panel. The optimal bread with large and medium fractions obtained the highest scores for overall acceptability, appearance, crumb structure, taste, and smell. Significant differences were observed between these two optimal bread samples and wheat flour bread.

### 3.4. Evaluating Relationships between Variables

**Figure 4:**
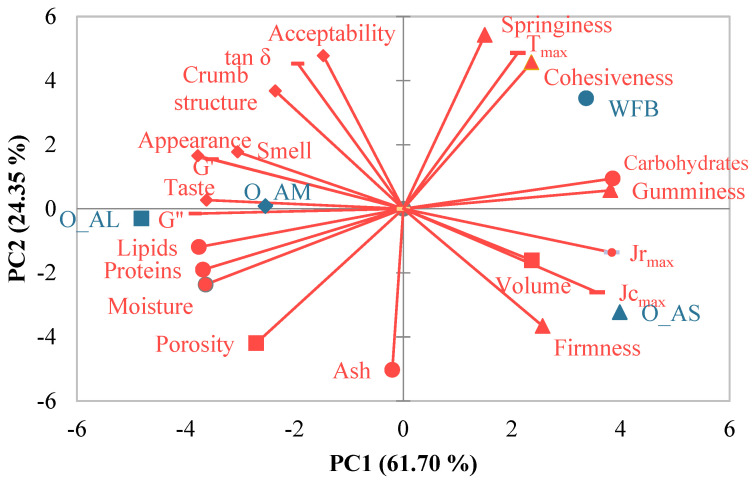
Principal component analysis bi-plot highlighting the relationships between the chemical compositions, dynamic dough rheological parameters, and the physical, textural, and sensory characteristics of breads with amaranth flour at optimal addition doses for each studied particle size. WFB—wheat flour bread; O_AL—optimal bread with large particle-size amaranth flour; O_AM—optimal bread with medium particle-size amaranth flour; O_AS—optimal bread with small particle-size amaranth flour.

Relationships between the variables were highlighted by applying a multivariate technique, principal components analysis (PCA). The application of PCA provided information about the similarities and/or differences between the evaluated variables, in terms of chemical composition, dough dynamic rheological parameters, bread physical, texture, and sensory properties (Figure 4).

From the analysis of Pearson correlation matrix, direct or indirect correlations were observed, significant at *p* < 0.05, between some of the determined characteristics.

## 4. Discussion

Considering the impact of the nutritional characteristics of wheat flour and amaranth flour fractions on the final product, the results of the mineral and amino-acids contents are discussed in relation to the nutritional properties of bread obtained from optimal wheat–amaranth composite flour specific to each particle size and wheat bread. Alencar et al. (2015) [35] found that adding 20% amaranth flour to a mixture containing rice flour, potato starch, cassava starch, and sour tapioca starch enhanced the nutritional value of bread due to the high levels of protein, fat, and minerals and a bread specific volume and firmness similar to control bread.

The increase in the FN index, of the optimal formulations with medium and small AF particle sizes, leads to a decrease in α-amylase activity, which can be correlated with the presence of calcium ions in amaranth grains [36]. Dough development time was correlated with dough strength and was significantly higher in the optimal composite flour, while dough stability didn’t present a difference compared with wheat flour. Starch retrogradation of the optimal composite flour showed significantly lower values than wheat flour, which can indicate that amaranth flour addition can limit starch retrogradation, increasing bread freshness and shelf-life. The biaxial extension of dough indicated a significant increase in the dough tenacity of the optimal composite sample, associated with lower extensibility. Fermentation properties were significantly improved for all of the optimal composite doughs, compared to the wheat flour dough. Dynamic rheological behavior indicated strong interactions between starch and gluten due to the high values of the G′ and G″ moduli. Moreover, the complex bonds that can be formed between starch granules and amaranth flour fibers can also determine a raise of the moduli. The variation of the viscosity factor (tan δ) for the optimal dough samples indicated a decrease e for the samples with medium and small particle sizes of amaranth flour (O_AL and O_AM), but all the optimal formulations showed values close to those of the control sample. This difference in viscosity factor values may be explained by the high protein content present in the amaranth flour fractions [27]. The maximum gelatinization temperature (T_max_) of optimal wheat–amaranth composite flour decreased in all of the optimal wheat–amaranth composite flours, a fact that may be due to the insoluble amylose–lipid complexes formation that occurred during the heating of starch slurries, which reduces and delays the swelling of starch granules [24]. Creep-recovery compliance is affected by the protein, carbohydrate, or starch content of amaranth flours. The hydroxyl groups will interact with proteins groups and will lead to non-covalent or covalent bonding [28]. The differences between the optimal formulations and the control samples in terms of empirical and dynamic rheological properties, as well as the volume and firmness of bread, can be explained by their contents of proteins, lipids, carbohydrates [27], minerals (Table S1), amino acids (Figure S1), and other compounds present in the amaranth flour fractions.

Bread obtained from optimal composite flour determined a slight decrease in volume. This behavior can be explained by the intake of dietary fiber from the large fractions, which interact with wheat glutenin through disulfide bonds, not diluting the gluten network very much [37]. The best results regarding bread porosity and elasticity were obtained when the large fraction was incorporated, this fact being possible due to the high content of starch and lipids from these fractions, which contribute as a stabilizing agent to the stabilization of the cells gases [38].

Regarding bread textural properties, an increase of crumb firmness in bread formulations was observed when AF particle size decreased. This fact can be explained by the dietary fiber content of the large particle, which contains albumin that can interact with wheat glutenin through disulfide bonds and will act as a surface active agent and thus will maintain the gluten matrix [37,39]. Similar results were obtained for breadspringiness, this phenomenon can be related to the high lipid content of the medium fraction of amaranth flour [27], as it is known that polar lipids can act as a gas stabilizer during the bread-making process, leading to an improvement in bread elasticity [38].

Generally, both colour of the crust and of the bread crumb impact consumer acceptability. The colour of the optimal bread formulations varied depending on the fraction used in optimal bread, this fact being possible due to the intense Maillard-type reaction that occurs in the samples with the addition of protein-rich fractions. In addition, the decrease in colour lightness of the optimal bread may also be explained by the higher amount of phenolic and flavonoids compounds of the AF [40,41]

The increase in the nutritional value of bread may be due to the high content of amaranth flour fractions in these nutrients [9], and the different proteins, lipids and ash localization in the seed. Data from the specialized literature show that amaranth seed proteins are located in the germ of the seed (65%) [42]. This variation is due to the different chemical compositions of the central endosperm, aleurone layer, embryo, and cell wall tissues in amaranth seeds, as explained by Steadman et al. (2001) [43].

The results obtained were consistent with those reported by Sanz-Penella et al. [13] and Miranda-Ramos et al. [44]. Bread with the addition of large, medium, and small particle sizes of AF at certain/optimal doses in wheat flour was enriched nutritionally, from a mineral point of view (Figure 4), compared to wheat bread. In general, white bread is low in minerals and could be supplemented to meet the daily requirements for different mineral elements [45,46], amaranth flour presenting high potential in this sense. Potassium (K) and calcium (Ca) were found in higher amounts in medium particles, while magnesium (Mg) and sodium (Na) were found in higher amount in small particles. For the total content of macro and micro elements, medium particles presented the highest content. For the small size fraction of amaranth flour, which consists mainly of the endosperm part of the seeds, a large amount of nutritionally important elements was found (Table S1). Minerals such as phosphorus, potassium, and magnesium are located in the embryo, while calcium is present in the pericarp and has been associated with those pectic compounds of the cell wall [47]. The cellular material in the ground endosperm can contribute significantly to the ash content [48]. Many of the minerals were identified in much higher concentrations than in other grains [49]. Considerable differences were observed between the optimal bread formulations and also between them and the wheat bread in terms of the determined minerals, but a high content of K, Ca, Mg, Zn, and Fe was found in the optimal samples compared to the control. The optimal bread with AF medium particle size presented the highest mineral content being followed by the bread with small particle size, results which were related to the minerals content from these fractions.

Miranda-Ramos et al. [44] replaced wheat flour with amaranth flour in doses of 25 and 50% and reported higher Ca, Fe, and Zn values for the formulated bread than those obtained in this research. Although Kumar et al. [50] studied the mineral content of whole AF and three fractions, to our knowledge, no other study has been conducted on the mineral content of bread with different AF particle sizes.

The bread made from optimal wheat–amaranth composite flour was enhanced with essential and nonessential amino acids compared to wheat flour bread. Methionine, phenylalanine, and threonine were the essential amino acids found in higher amounts in the optimal bread for all amaranth fractions. The variation in the amino acids content can be explained due to the presence of the AA from the amaranth fractions (Figure S1) that are present especially in the bran, which creates an intercellular skeleton that prevents the action of digestive enzymes and thus reduces the content of amino acids in the seed coat [51]. Asparagine was not found, because it was completely converted to aspartic acid under acid hydrolysis conditions [52]. Aspartic and glutamic acids were present in larger quantities in all of the optimal bread formulations, glutamic acid being present in larger quantities than in the control bread only in the optimal bread with small size fractions (O_AS).

One of the main challenges in wheat flour bread production replaced by non-gluten amaranth flour is the level of consumer acceptance. This is crucial considering that the appearance, aroma, taste, and texture of food play a key role in the acquisition process. Compared with the control sample, an improvement of the organoleptic characteristics of the optimal bread formulations, excepting optimal bread with AF small particle size, was found. The optimal bread with medium and large particle sizes presented similar or better sensorial characteristics than that of the control bread. The respondent’s acceptance can be explained by the soft crumbs due to the presence of natural emulsifiers in amaranth flours. Our results are in agreement with those found by other authors when they made bread from amaranth flour [53].

The use of principal component analysis (PCA) highlighted a plot of the features and type of the sample on the two principal components (Figure 4). The first two main components (PC1 and PC2) explained 86.05% of the total variance of the analyzed variables, where PC1 explained 61.70%. PC1 was associated with the viscoelastic moduli and the maximum creep and recovery compliance of dough samples as well as with the moisture, protein, lipid, and carbohydrate contents of optimal breads and the firmness, gumminess, porosity, appearance, crumb structure, taste, and smell of bread samples. PC2 was associated with the viscosity factor and the maximum gelatinization temperature of the dough, ash content, springiness, cohesiveness, and overall acceptability of bread. A strong association was observed between the optimal composite flour with large PS and the lipid, protein, and porosity of the bread. The optimal sample with medium particles of amaranth flour was associated with viscoelastic moduli and bread taste, while the optimal sample with small-PS amaranth flour was related to the maximum creep and recovery compliance of the dough and with the bread volume and firmness. By applying the Pearson correlation analysis between the evaluated characteristics, a series of very strong correlations were obtained (0.95 < r < 0.99), significant at a significance level of *p* < 0.05. The protein content of bread was directly correlated with the viscosity modulus (r = 0.95) and inversely with the gumminess and chewiness of the bread (r = −0.97). Positive correlations were obtained between the carbohydrate content of bread and the gumminess (r = 0.99) and chewiness (r = 0.99), while these texture parameters are negatively associated with the viscosity modulus (r = −0.98; r = −0.99). Regarding bread firmness, an inverse correlation was found with the viscosity factor (r = −0.97), while bread elasticity was directly associated with the maximum gelatinization temperature (r = 0.98). Bread cohesiveness was negatively associated with bread crumb porosity (r = −0.99). The appearance of the bread was negatively correlated with the bread volume (r = −0.96), while the taste of the bread was positively correlated with the viscous modulus (r = 0.98). Some authors found a direct correlation between dough elasticity and bread firmness and chewiness [54,55].

## 5. Conclusions

The bread produced from optimal wheat–amaranth composite flour specific to each particle size presented an improvement in volume, porosity, and elasticity, while the crust and crumb lightness, firmness, resilience, and chewiness decreased in bread with small particle sizes compared to wheat-flour bread. From a nutritional point of view, bread made with amaranth flour at an established addition dose depending on particle size showed a significant increase in protein, lipid, and ash content, with higher content of potassium, calcium, magnesium, zinc, and iron compared to the wheat flour bread values, while the carbohydrate content and energy value decreased. The highest protein content was found in bread with medium and small particle sizes, an increase of 26.7%, and 15.8%, respectively, being observed. There was an increase in the mineral content by over 37% in the bread samples obtained with composite flour with medium and small particles, and an improvement in protein by more than 26% in the optimal bread with large particles compared to wheat flour bread. Phenylalanine, threonine, and valine were the essential amino acids, while aspartic and glutamic acids were the nonessential amino acids that were identified in higher amounts in the studied samples. An increase in the total amino acid content of 19.5% and 8.7% was observed in the optimal bread with small and medium amaranth flour, respectively. The results of the sensory analysis revealed an increase in the overall acceptability score, appearance, crumb structure, taste, and smell for the bread samples obtained from the optimal composite flours containing medium and large particles of amaranth flour. The bread made from composite flour with medium particles was very well accepted with a score higher than 8.5 for the general acceptability.

As a result, it can be concluded that the optimal composite bread with AF medium particle size presents the highest nutritional, physical, and sensorial values, followed by the optimal composite bread made with AF small particle size.

## Figures and Tables

**Table 1:** The characteristics of the optimal wheat–amaranth composite flour for each amaranth flour particle size, dough rheology, and the bread parameters as compared to wheat dough bread.

Characteristic	WF	O_AL	O_AM	O_AS
Addition dose (%)	100	9.41	9.39	7.89
FN (s)	312.00 ± 5.25 ^axA^	312.22 ± 3.78 ^a^	317.03 ± 6.11 ^y^	315.85 ± 4.65 ^B^
WA (%)	58.50 ± 0.02 ^axA^	57.94 ± 0.58 ^a^	58.68 ± 0.34 ^x^	59.52 ± 0.03 ^A^
DT (min)	1.69 ± 0.75 ^axA^	3.08 ± 0.75 ^b^	3.00± 1.05 ^y^	3.05 ± 0.90 ^B^
ST (min)	9.96 ± 0.65 ^axA^	9.95 ± 0.66 ^a^	9.50 ± 0.70 ^x^	10.54 ± 0.70 ^A^
C1-2 (N∙m)	0.61 ± 0.02 ^axA^	0.54 ± 0.01 ^a^	0.60 ± 0.02 ^x^	0.62 ± 0.02 ^A^
C3-2 (N∙m)	1.41 ± 0.03 ^axA^	1.30 ± 0.02 ^a^	1.30 ± 0.04 ^x^	1.31 ± 0.03 ^A^
C3-4 (N∙m)	0.05 ± 0.04 ^axA^	0.20 ± 0.04 ^b^	0.165 ± 0.03 ^y^	0.10 ± 0.01 ^A^
C5-4 (N∙m)	1.15 ± 0.01 ^bxA^	0.88 ± 0.05 ^a^	0.91 ± 0.11 ^x^	0.85 ± 0.01 ^A^
P (mm H_2_O)	87.00 ± 5.75 ^axA^	91.50 ± 3.70 ^b^	92.88 ± 7.76 ^y^	95.05 ± 5.40 ^B^
L (mm)	91.00 ± 10.50 ^bxA^	51.47 ± 3.04 ^a^	58.93 ± 6.37 ^x^	55.25 ± 14.85 ^B^
W (10^−4^ J)	253.00 ± 20.14 ^byA^	180.00 ± 11.91 ^a^	173.60 ± 24.54 ^x^	180.42 ± 18.65 ^B^
P/L (adim.)	0.95 ± 0.05 ^axA^	1.70 ± 0.25 ^b^	1.77 ± 0.54 ^y^	2.02 ± 0.50 ^B^
H′m (mm)	62.00 ± 4.25 ^axA^	75.27 ± 5.20 ^b^	75.58 ± 3.86 ^y^	70.95 ± 2.85 ^B^
VT (mL)	1168.00 ± 89.56 ^axA^	1260.57 ± 19.81 ^a^	1290.19 ± 58.38 ^y^	1197.50 ± 17.25 ^B^
VR (mL)	991.20 ± 85.25 ^axA^	1160.83 ± 3.74 ^a^	1158.70 ± 36.53 ^y^	1195.45 ± 96.45 ^B^
CR (%)	84.20 ± 2.50 ^axA^	90.86 ± 2.77 ^b^	89.32 ± 1.62 ^y^	89.75 ± 5.45 ^B^
G′ (Pa)	26,370.00 ± 10.00 ^bxA^	28,901.40 ± 287.25 ^b^	26,235.03 ± 95.39 ^y^	22,320.25 ± 10.25 ^A^
G″ (Pa)	9488.00 ± 74.58 ^bxA^	11,413.4 ± 691.37 ^b^	9975.05 ± 869.51 ^y^	102,560.05 ± 8.69 ^B^
tan δ (adim.)	0.360 ± 0.02 ^axA^	0.352 ± 0.01 ^a^	0.345 ± 0.02 ^x^	0.344 ± 0.02 ^A^
Tmax (°C)	83.24 ± 0.55 ^bxA^	80.47 ± 0.45 ^a^	80.56 ± 0.96 ^x^	79.58 ± 0.45 ^A^
Jcmax (10^−5^ Pa^−1^)	24.50 ± 4.50 ^axA^	20.58 ± 2.54 ^a^	20.24 ± 5.31 ^x^	25.45 ± 3.47 ^A^
Jrmax (10^−5^ Pa^−1^)	16.62 ± 2.40 ^axA^	13.11 ± 1.45 ^a^	17.42 ± 3.04 ^y^	17.95 ± 2.63 ^B^
BV (cm^3^)	372.20 ± 15.25 ^bxA^	349.10 ± 6.71 ^a^	372.92 ± 14.04 ^x^	341.77 ± 1.50 ^A^
BF (N)	7.55 ± 3.00 ^axA^	9.61 ± 0.71 ^a^	6.97 ± 3.10 ^x^	12.55 ± 10.52 ^B^

WF—wheat flour; O_AL—optimal composite flour with large particle-size amaranth flour; O_AM—optimal composite flour with medium particle-size amaranth flour; O_AS—optimal composite flour with small particle-size amaranth flour. Mean values on the same row followed by different letters are significantly different (*p* < 0.05): for the differences between the control and optimal formulations values. FN—Falling number; WA—water absorption capacity; DT—development time; ST—dough stability; C1-2—protein denaturation; C3-2—starch gelatinization; C3-4—stability of hot starch gel; C5-4—starch retrogradation; P—tenacity; L—extensibility; W—deformation energy; P/L—alveographic ratio; H’m—maximum height; VT—total volume of gas; VR—volume of gas retained; CR—gas retention coefficient; G′—modulus of elasticity; G"—viscosity modulus; tan δ—viscosity factor; T_max_—maximum gelatinization temperature; Jc_max_—maximum creep compliance; Jr_max_—maximum recovery compliance; BV—bread volume; BF—bread firmness.

**Table 2:** Physical parameters of breads with the optimal dose (O) corresponding to large, medium, and small particle sizes of amaranth flour (AL, AM, and AS) compared to wheat flour bread (WFB).

Bread Sample	Specific Volume (cm^3^/g)	Porosity (%)	Elasticity (%)
WFB	2.45 ± 0.25 ^b^	64.22 ± 5.62 ^c^	91.70 ± 6.52 ^d^
O_AL	2.55 ± 0.50 ^a^	70.48 ± 4.75 ^a^	95.91 ± 8.52 ^a^
O_AM	2.47 ± 0.27 ^b^	67.33 ± 7.28 ^b^	94.98 ± 2.45 ^b^
O_AS	2.35 ± 0.85 ^c^	66.89 ± 5.62 ^c^	94.32 ± 8.45 ^c^

WFB—wheat flour bread; O_AL—optimal bread with large particle size amaranth flour; O_AM—optimal bread with medium particle size amaranth flour; O_AS—optimal bread with small particle-size amaranth flour. Mean values on the same column followed by different letters are significantly different (*p* < 0.05).

**Table 3:** Colour parameters of bread samples with the optimal dose (O) corresponding to large, medium, and small particle sizes of amaranth flour (AL, AM, and AS) compared to wheat flour bread (WFB).

Bread Sample	Crust Colour Parameters			Crumb Colour Parameters		
	L*	a*	b*	L*	a*	b*
WFB	70.35 ± 0.91 ^a^	−1.33 ± 0.22 ^d^	32.27 ± 0.28 ^c^	73.94 ± 0.27 ^a^	−4.48 ± 0.03 ^b^	20.02 ± 0.23 ^a^
O_AL	66.8 ± 1.07 ^b^	2.40 ± 0.16 ^c^	34.33 ± 0.39 ^b^	63.05 ± 0.46 ^b^	−3.76 ± 0.02 ^ab^	19.94 ± 0.54 ^a^
O_AM	62.46 ± 0.80 ^c^	5.89 ± 0.29 ^a^	37.02 ± 0.27 ^a^	63.70 ± 0.98 ^b^	−3.37 ± 0.22 ^a^	21.46 ± 0.54 ^a^
O_AS	64.68 ± 0.87 ^bc^	3.47 ± 0.29 ^b^	34.94 ± 0.29 ^b^	65.57 ± 0.38 ^b^	−4.18 ± 0.42 ^ab^	20.32 ± 0.43 ^a^

WFB—wheat flour bread; O_AL—optimal bread with large particle-size amaranth flour; O_AM—optimal bread with medium particle-size amaranth flour; O_AS—optimal bread with small particle-size amaranth flour. L*, a*, b*—the degree of lightness, the intensity of red or green and, respectively, of yellow or blue. Mean values on the same column followed by different letters are significantly different (*p* < 0.05).

**Table 4:** Texture parameters of bread samples with the optimal dose (O) corresponding to the large, medium, and small particle sizes of amaranth flour (Al, AM, and AS) compared to those of wheat flour bread (WFB).

Bread Sample	Springiness (Adim.)	Cohesiveness (Adim.)	Gumminess (N)	Resilience (Adim.)	Masticability (N)
WFB	1.3457 ± 0.27 ^a^	0.8575 ± 0.01 ^a^	499.73 ± 4.63 ^a^	1.8278 ± 0.00 ^ab^	499.73 ± 4.63 ^a^
O_AL	1.0740 ± 0.10 ^c^	0.8202 ± 0.01 ^b^	455.43 ± 41.67 ^a^	1.6270 ± 0.00 ^b^	455.43 ± 41.67 ^a^
O_AM	1.0000 ± 0.00 ^b^	0.8264 ± 0.02 ^b^	511.96 ± 11.56 ^a^	2.1453 ± 0.49 ^a^	511.96 ± 11.56 ^a^
O_AS	1.1200 ± 0.03 ^b^	0.8314 ± 0.00 ^b^	498.42 ± 35.62 ^a^	2.0295 ± 0.06 ^a^	498.42 ± 35.62 ^a^

WFB—wheat flour bread; O_AL—optimal bread with large particle-size amaranth flour; O_AM—optimal bread with medium particle-size amaranth flour; O_AS—optimal bread with small particle-size amaranth flour. Mean values on the same column followed by different letters are significantly different (*p* < 0.05).

**Table 5:** Physico-chemical properties of breads with the optimal dose (O) corresponding to large, medium, and small particle sizes of amaranth flour (Al, AM, and AS) compared to to those of wheat flour bread (WFB).

Bread Sample	Moisture (%)	Proteins (%)	Lipids (%)	Ash (%)	Carbohydrates (%)	Energetic Value (kcal)
WFB	43.12 ± 0.03 ^d^	8.35 ± 0.13 ^bc^	0.01 ± 0.00 ^b^	0.72 ± 0.02 ^b^	47.81 ± 0.11 ^a^	230.31 ± 0.22 ^a^
O_AL	44.20 ± 0.07 ^a^	10.58 ± 0.12 ^a^	0.17 ± 0.02 ^a^	0.82 ± 0.02 ^b^	44.23 ± 0.04 ^c^	226.32 ± 0.51 ^b^
O_AM	43.95 ± 0.03 ^b^	9.67 ± 0.06 ^b^	0.17 ± 0.02 ^a^	0.99 ± 0.01 ^a^	45.23 ± 0.07 ^b^	226.65 ± 0.23 ^b^
O_AS	43.50 ± 0.03 ^c^	8.92 ± 0.12 ^c^	0.04 ± 0.02 ^b^	0.99 ± 0.01 ^a^	46.55 ± 0.20 ^a^	227.75 ± 0.31 ^b^

WFB—wheat flour bread; O_AL—optimal bread with large particle-size amaranth flour; O_AM—optimal bread with medium particle-size amaranth flour; O_AS—optimal bread with small particle-size amaranth flour. Mean values on the same column followed by different letters are significantly different (*p* < 0.05).

## Data Availability

Data is contained within the article.

## Supplementary Materials

The following supporting information can be downloaded at: https://www.mdpi.com/article/10.3390/foods12010133/s1, Table S1: The mineral content of wheat flour (WF) compared to the large (L), medium (M) and small (S) particle size of amaranth flour; Figure S1: Essential (a) and non-essential (b) amino acid content of amaranth flour (AF) corresponding to large (L), medium (M) and small (S) particle size compared to wheat flour.

## Abbreviations

WF	wheat flour
AF	amaranth flour
O_AL	optimal composite flour with large particle-size amaranth flour
O_AM	optimal composite flour with medium particle-size amaranth flour
O_AS	optimal composite flour with small particle-size amaranth flour
