# Supplementary material for: Addition of Amaranth Flour of Different Particle Sizes at Established Doses in Wheat Flour to Achieve a Nutritional Improved Wheat Bread
Source: Foods. 2022 Dec 27;12(1):133. doi: 10.3390/foods12010133 (PMC9818772; doi:10.3390/foods12010133)
Supplement: Supplementary file 1 [file foods-12-00133-s001.zip › foods-2125799-supplementary.pdf]

Table S1. The mineral content of wheat flour (WF) compared to the large (L), medium (M) and small (S) particle size of amaranth flour

Parameter	WF	Amaranth flour particle size		
		AL	AM	AS
**Macro-elements (mg/100 g)**				
K	108.50 ± 2.12^{e}	295.10 ± 0.56^{c}	507.05 ± 18.31^{a}	360.05 ± 14.14^{b}
Ca	24.80 ± 0.10^{d}	83.03 ± 0.61^{c}	90.20 ± 2.72^{c}	197.66 ± 0.38^{a}
Mg	155.5 ± 0.65^{b}	159.8 ± 0.04^{a}	165.0 ± 0.26^{a}	166.00 ± 0.11^{a}
Na	7.33 ± 0.11^{b}	10.94 ± 1.04^{a}	11.42 ± 1.63^{a}	11.88 ± 1.14^{a}
**Micro-elements (mg/100 g)**				
Fe	1.80 ± 0.06^{e}	2.95 ± 0.47^{c}	5.71 ± 0.98^{b}	5.73 ± 0.44^{b}
Zn	3.02 ± 0.25^{b}	5.88 ± 0.13^{a}	6.15 ± 0.34^{a}	6.49 ± 0.39^{a}
Mn	1.59 ± 0.10^{e}	1.80 ± 0.18^{c}	2.89 ± 0.68^{bc}	4.59 ± 0.52^{ab}
Cu	0.56 ± 0.01^{e}	0.62 ± 0.02^{bc}	0.81 ± 0.04^{ab}	0.95 ± 0.08^{a}

WF – wheat flour; AL – large particle size of amaranth flour; AM – medium particle size of amaranth flour; AS – small particle size of amaranth flour. Mean values on the same column followed by different letters are significantly different ($p < 0.05$).

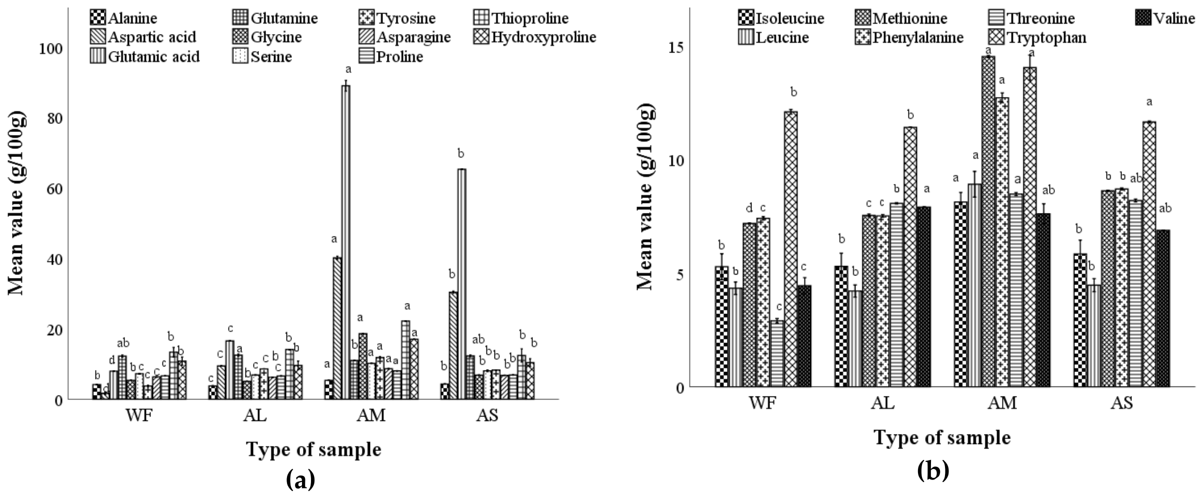

Figure S1. Essential (a) and non-essential (b) amino acid content of amaranth flour (AF) corresponding to large (L), medium (M) and small (S) particle size compared to wheat flour
